# Epidemiologic characteristics and clinical outcomes of respiratory syncytial virus in hospitalized care in Lebanon: a prospective observational study

**DOI:** 10.3389/fcimb.2025.1711410

**Published:** 2026-01-07

**Authors:** Sarah Khafaja, Sarah Merhi, Stephanie Damaj, Saja Issaoui, Celina F. Boutros, Habib Al-Kalamouni, Nadia Soudani, Yolla Youssef, Zeinab El-Zein, Samer Bou-Karroum, Ahmad Chmaisse, Magda Haj, Zeina Houry, Sarah Chamseddine, Nour Youssef, Rouba Shaker, Amal Naous, Soha Ghanem, Chantale Lahoud, Imad Shokr, Rita Feghali, David Breish, Amani Haddara, Maria Karam, Hassan Zaraket, Ghassan S. Dbaibo

**Affiliations:** 1 Center for Infectious Diseases Research (CIDR) and WHO Collaborating Center for Reference and Research on Bacterial Pathogens, Faculty of Medicine, American University of Beirut, Beirut, Lebanon; 2 Department of Pediatrics and Adolescent Medicine, American University of Beirut Medical Center, Beirut, Lebanon; 3 Pediatric Infectious Diseases Division, Department of Pediatrics and Adolescent Medicine, American University of Beirut Medical Center, Beirut, Lebanon; 4 Department of Experimental Pathology, Immunology, and Microbiology, Faculty of Medicine, American University of Beirut, Beirut, Lebanon; 5 Department of Pediatrics, New Mazloum Hospital, Tripoli, North Lebanon, Lebanon; 6 Department of Pediatrics, Makassed General Hospital, Beirut, Lebanon; 7 Department of Pediatrics, Rafic Hariri University Hospital, Beirut, Lebanon; 8 Department of Laboratory Medicine, Rafic Hariri University Hospital, Beirut, Lebanon; 9 Department of Pediatrics, Bekaa Hospital, Bekaa, Lebanon; 10 Department of Pediatrics, Keserwan Medical Center, Jounieh, Mount Lebanon, Lebanon

**Keywords:** adults, children, epidemiology, Lebanon, respiratory syncytial virus, risk factors, severity outcomes

## Abstract

**Introduction:**

The aim of this study was to describe the epidemiology and clinical outcomes of respiratory syncytial virus (RSV) infection in hospitalized children and adults from a sentinel surveillance network in Lebanon, and to compare the RSV rates and seasonality before and during COVID-19 pandemic.

**Methods:**

This study was based on the data from the Lebanese component of the Global Influenza Hospital Surveillance Network (GIHSN) during three consecutive seasons between 2019–2021 at six hospitals, following a standardized protocol based on age-specific criteria. Specimens were tested for RSV. The epidemiologic, clinical, and severity characteristics were analyzed. Descriptive statistics were used to summarize demographics, RSV rate, and outcomes. Univariate analyses were performed using Pearson’s Chi-square or Fisher’s exact test, and associations were expressed as unadjusted odds ratios (95% CI). A p-value ≤0.05 was considered significant.

**Results:**

From January 2019 to September 2021, 2,626 of 7,081 eligible inpatients were enrolled in the study, with 188 tested positives for RSV (7.3%). The majority (74.5%) of RSV-positive subjects were children under 5 years of age, and 9.6% of patients were ≥ 65 years of age. The positivity rate varied across seasons (2.1%-11.1%) (p-value <0.001); the 2020–2021 season —was disrupted by COVID-19 pandemic — recording the lowest rate of 1.1% (p-value <0.001) during the typical winter months followed by an off-season RSV resurgence. Fever, cough, nasal congestion, wheezing, neurological symptoms, diarrhea and decreased oral intake were significantly associated with RSV infection (p < 0.05). Cough and wheezing were predominant in RSV-positive children <5 and adults aged ≥ 65 years. Among RSV-positive subjects, 8% required ICU admission, 5.3% received mechanical ventilation, and 2.7% died. Severe outcomes were more common in those ≥65 years, and the presence of ≥2 comorbidities and underlying cardiovascular disease significantly increased the risks of mechanical ventilation (OR = 7.44 [1.13-48.99], p-value 0.037 and OR = 8.32 [2.20-31.37], p-value 0.004, respectively) and in-hospital death (OR = 22.67 [1.91-268.50], p-value 0.013 and OR = 11.50 [1.82-72.85], p-value 0.016, respectively).

**Conclusions:**

This study demonstrates the significant burden of RSV, especially in young infants with or without co-morbidities and in older adults with co-morbidities. Given the recent rollout of RSV vaccines and monoclonal antibodies, continued surveillance is needed to monitor changes in epidemiology, seasonality, and disease burden, especially in low- and middle-income countries.

## 1 Introduction

Respiratory Syncytial Virus (RSV) is a leading cause of acute lower respiratory infections (ALRI) in infants and children throughout the world, resulting in annual epidemics (Tabor et al., 2020; WHO, 2024). Children under 5 years of age are the most affected age group (World Health Organization (WHO), 2025). Recently, it is increasingly recognized as a major contributor to respiratory illnesses among adults, leading to an increase in hospitalization rates, especially among those aged 65 years and above (WHO, 2024).

Globally in 2019, it was estimated that RSV was associated with 33.0 million episodes of ALRI, 3.6 million hospital admissions, and 101,400 deaths in children less than 5 years of age (Li et al., 2022). Approximately 39% of hospital admissions and 50% of in-hospital deaths due to RSV-ALRI occurred in children younger than 6 months, and more than 97% of RSV-attributable deaths across all age bands were in low- and middle-income countries (LMICs) (Li et al., 2022). A recent systematic review conducted by our group on the burden of RSV infection in the Middle East and North Africa (MENA) region across age groups highlighted the scarcity of data related to RSV disease from LMICs particularly in adult patients (Youssef et al., 2021).

RSV infections have a higher burden in children with preexisting medical conditions such as preterm birth, chronic lung disease, congenital heart disease, Down syndrome, and other rare congenital/metabolic diseases (Langley and Anderson, 2011). In addition, a systematic review published in 2015 showed that prematurity, low birth weight, male gender, having siblings, maternal smoking, history of atopy, no breastfeeding and crowding were significant risk factors for RSV-associated ALRI in children under 5 years of age (Shi et al., 2015). Moreover, a systematic review of RSV in adults found greater morbidity and mortality related to RSV infections in transplant and immunocompromised patients, and in patients with chronic respiratory and congestive heart diseases (Tin Tin Htar et al., 2020).

RSV is known to cause seasonal outbreaks that occur over different periods of the year depending on the climate and geographic location. Analysis of surveillance data in 27 countries revealed consistent seasonal patterns over time with peak activity during the winter months. In the Southern Hemisphere RSV activity started between March and June and moved to the Northern Hemisphere between September and December (Obando-Pacheco et al., 2018; Chadha et al., 2020). In contrast, in the tropical regions a wide variability in the timing and duration of epidemics exists, with virus activity being observed throughout the year with a peak during the rainy season (Bloom-Feshbach et al., 2013; Obando-Pacheco et al., 2018).

Many countries monitor RSV as part of their influenza surveillance system (Obando-Pacheco et al., 2018). Nonetheless, in the MENA region, there are few centers capable of conducting comprehensive surveillance for RSV on a national or sentinel scale, with the capacity to thoroughly investigate other viruses, including molecular diagnosis and typing, viral culture, sequencing, and determining antiviral resistance. Understanding the regional and local trends and epidemiology of RSV is critical to inform the implementation of preventive and prophylactic measures especially as a growing number of vaccines and prophylactic monoclonal antibodies are becoming available (Obando-Pacheco et al., 2018; Venkatesan, 2023; Günther and Sandmann, 2024).

Therefore, in this study we aimed to describe the epidemiology and burden of RSV infection in hospitalized children and adults between January 2019 and September 2021, using the data collected in Lebanon as part of the Global Influenza Hospital Surveillance Network (GIHSN), and to compare the RSV rates before and during the COVID-19 pandemic. Moreover, this study aimed to assess the outcomes and severity of RSV infections and to determine the associated risk factors for severe disease.

## 2 Materials and methods

### 2.1 Study design

GIHSN is a global multicenter, prospective, active-surveillance, hospital-based influenza epidemiological observational network, which was established to collect epidemiological and virological data on influenza from both hemispheres. This network includes more than 100 hospitals in more than 25 countries performing active surveillance of influenza, following the same core investigation protocol (GIHSN, 2024; GIHSN, 2021). The Center for Infectious Diseases Research (CIDR), based at the American University Beirut Medical Center (AUBMC) in Lebanon and a member of GIHSN since 2019, is one of the few centers in the MENA region that has the capacity of conducting comprehensive surveillance for RSV and other respiratory viruses (GIHSN, 2023). During the period from January 2019 to September 2021, respiratory specimens and their data were collected from six participating hospitals located in different governorates in Lebanon and constituting the Lebanese sentinel surveillance network of GIHSN: AUBMC, Rafic Hariri University Hospital and Makassed General Hospital from Beirut, Keserwan Medical Center from Mount Lebanon, New Mazloum hospital from North Lebanon, and Beqaa Hospital from Beqaa.

### 2.2 Population

#### 2.2.1 Eligibility and inclusion criteria

Patients of all age groups who had been hospitalized within the previous 72 hours and had spent at least one night in the hospital were eligible if they met a broad predefined set of conditions (admission diagnoses), including acute respiratory disease, cardiac disease, metabolic failure, gastrointestinal manifestations, and others (**Supplementary Tables S1**, **S2**). Each morning, admission registries from the preceding 24 hours (72 hours on weekends) were reviewed. Admission diagnoses were screened using the standardized age-specific criteria: **Supplementary Table S1** for patients aged ≥5 years and **Supplementary Table S2** for children <5 years. Only patients fulfilling one of these clinical case definitions were approached and considered eligible for enrollment. The active screening process was conducted by research fellows, physicians, and study nurses. Eligible patients were approached, and the details of the study and sample collection techniques were explained to them. A written informed consent was signed if they accepted to be enrolled in the study. Patients who were 5 years of age or older were included if they met the combined criteria based on the European Center for Disease Control and Prevention modified case definition for influenza like-illness (ILI) in the previous 7 days (at least one of the four systemic symptoms (fever or feverishness, headache, myalgia or malaise) plus at least one of the four respiratory symptoms (cough, sore throat, shortness of breath or nasal congestion. Patients younger than 5 years of age were included if any of these symptoms occurred within seven days or less before admission to the hospital.) (GIHSN, 2021; Copenhagen: WHO Regional Office for Europe and Stockholm: European Centre for Disease Prevention and Control, 2022).

#### 2.2.2 Exclusion criteria

Patients unable to communicate or consent to participation and those with a history of hospitalization within the previous 30 days prior to recruitment were excluded from the study.

### 2.3 Data collection

After signing the informed consent, the study personnel completed a case report form. Baseline and demographic data were collected including age, gender, weight, residence area, household crowding index, school or daycare attendance, workplace, smoking habits (for patients between 5 and 13 years old, the answer referred to the smoking habits of the parents/tutors/household members. For patients 14 years old or more, it described the smoking habits of the patient), comorbidities (cardiovascular disease/high blood pressure, chronic obstructive pulmonary disease, asthma, diabetes mellitus, immunodeficiency (except HIV)/organ transplant, renal disease, rheumatologic disease/autoimmune disease, neurological or neuromuscular disease, cirrhosis/liver disease, active neoplasm. Obesity, malnutrition (only for children < 5years), active tuberculosis, HIV infection/exposure, leukemia, hemoglobinopathy, prematurity (only for children < 5years), and others if present) use of any antiviral or antibiotic before admission, presence of sick contacts, and travel history. In addition, the influenza vaccination history for the current season, the date of vaccination and type of vaccine received (trivalent or quadrivalent) were collected. Medical records were reviewed for laboratory proven bacterial co-infection, severity, and vital signs at admission, during admission, complications during hospital stay, severity at any time of admission (ICU admission, mechanical ventilation requirement, use of vasopressors) and outcome (recovery and discharge, in-hospital death, transfer to another facility, or leaving against medical advice). Data collection and entry were performed at CIDR.

### 2.4 Sample collection and storage

For all enrolled subjects before November 2020, samples were collected as follows: nasopharyngeal and pharyngeal swabs from those who were ≥ 14 years old and nasal swabs from those who were < 14 years old. Starting November 2020, all patients admitted to the hospital were tested for SARS-CoV-2 using real-time Polymerase Chain Reaction (RT-PCR) at each hospital per local protocol. Therefore, for patients with confirmed positive or negative SARS-CoV-2 test, an additional pharyngeal swab was collected if the patient accepted. For those who refused to repeat nasopharyngeal/nasal swabbing, the leftover sample collected on admission was retrieved from the laboratory. Samples were preserved in viral transport media (VTM), stored at 4°C, and transferred to the CIDR at AUBMC within 72 hours of collection or kept at -20°C in case shipment to the laboratory was expected to take more than 72 hours. Samples were further analyzed in line with the required proper packaging for time- and temperature- sensitive hazardous samples. Those cases were then tested for influenza and RSV at the CIDR laboratory.

### 2.5 Laboratory analysis

In addition to testing for influenza (AlIbrahim et al., 2019; Assaf-Casals et al., 2020), all samples were tested for RSV. For RSV testing, samples were aliquoted as follows: RNA extraction was performed using the Purelink viral RNA/DNA Mini Kit (Thermo Fisher Scientific) following the manufacturer’s instruction (Bashir et al., 2013). After extraction, 4 µl of the RNA extract underwent probe-based quantitative reverse-transcription polymerase chain reaction (rt-qPCR) (Agpath-ID-Applied Biosystem) to detect the presence of RSV in the samples using a pair of primers that target the conserved region of the viral matrix gene (**Table 1**).

**Table 1:** Laboratory analysis for the detection of RSV using a pair of primers.

Primer	Sequence
RSV Forward	GGCAAATATGGAAACATACGTGAA
RSV Reverse	TCTTTTTCTAGGACATTGTAYTGAACAG
RSV Probe	CTGTGTATGTGGAGCCTTCGTGAAGCT

### 2.6 Definitions

RSV season is defined as spanning from October 1^st^ of a given year to September 30^th^ of the following year. In the present study, recruitment during the first season 2018–2019 started in January 2019 due to some delays in study approval. The surveillance was stopped from June 2019 to September 2019 and from June 2020 till September 2020, in accordance with the initial GIHSN protocol. In addition, recruitment was stopped from January 11, 2021, until March 8, 2021, due to some restrictions imposed by the Institutional Review Board (IRB) regarding human subject research during the lockdown period.

The pre-pandemic period was defined as beginning of 2018/2019 season until the global pandemic was declared by the World Health Organization on March 11, 2020 (Cucinotta and Vanelli, 2020).

Severe RSV disease was defined as that requiring mechanical ventilation, admission to ICU or resulting in a fatal outcome.

### 2.7 Ethical considerations

This research was approved by the AUB IRB as “Global Influenza Hospital Surveillance Network-Lebanon” (protocol number: BIO-2018-0501) in compliance with the World Medical Association Declaration of Helsinki in 2013. After we explained the purpose of the study and the importance of participation, a written consent from the subject/legal representative or witness was essential to proceed with the data and samples collection. Participants were also informed that there were no risks or direct benefits from their collaboration on this study. The participation was completely voluntary, and enrolled subjects retained the right to withdraw at any time throughout the study. In addition, to maintain confidentiality, all data were kept anonymous in the interview questionnaire, after which they will be destroyed once the legal retention period expires.

### 2.8 Statistical analysis

Collected data were coded and entered in the software Statistical Package for Social Sciences (SPSS) version 29 (SPSS™ Inc., Chicago, IL United States). Descriptive statistics were conducted to analyze the demographic characteristics of subjects, disease outcomes, RSV incidence, and temporal dynamics of RSV. Moreover, univariate analysis of risk factors for RSV infection were analyzed by Pearson’s Chi Square test or Fischer’s exact test (when number of subjects in a subgroup is less than 5). Continuous risk factors were analyzed with the student t test. Statistical significance is considered below type-1 error threshold (alpha level) of 0.05. The strength of association was interpreted using the Unadjusted Odds Ratio (UOR) with 95% Confidence Interval (CI). p ≤ 0.05 was considered statistically significant. Further analyses of the complications and severity factors of RSV infection including ICU admission, oxygen supplementation, need for mechanical ventilation and mortality were performed.

## 3 Results

### 3.1 RSV detection and co-infection rates

**Figure 1:**
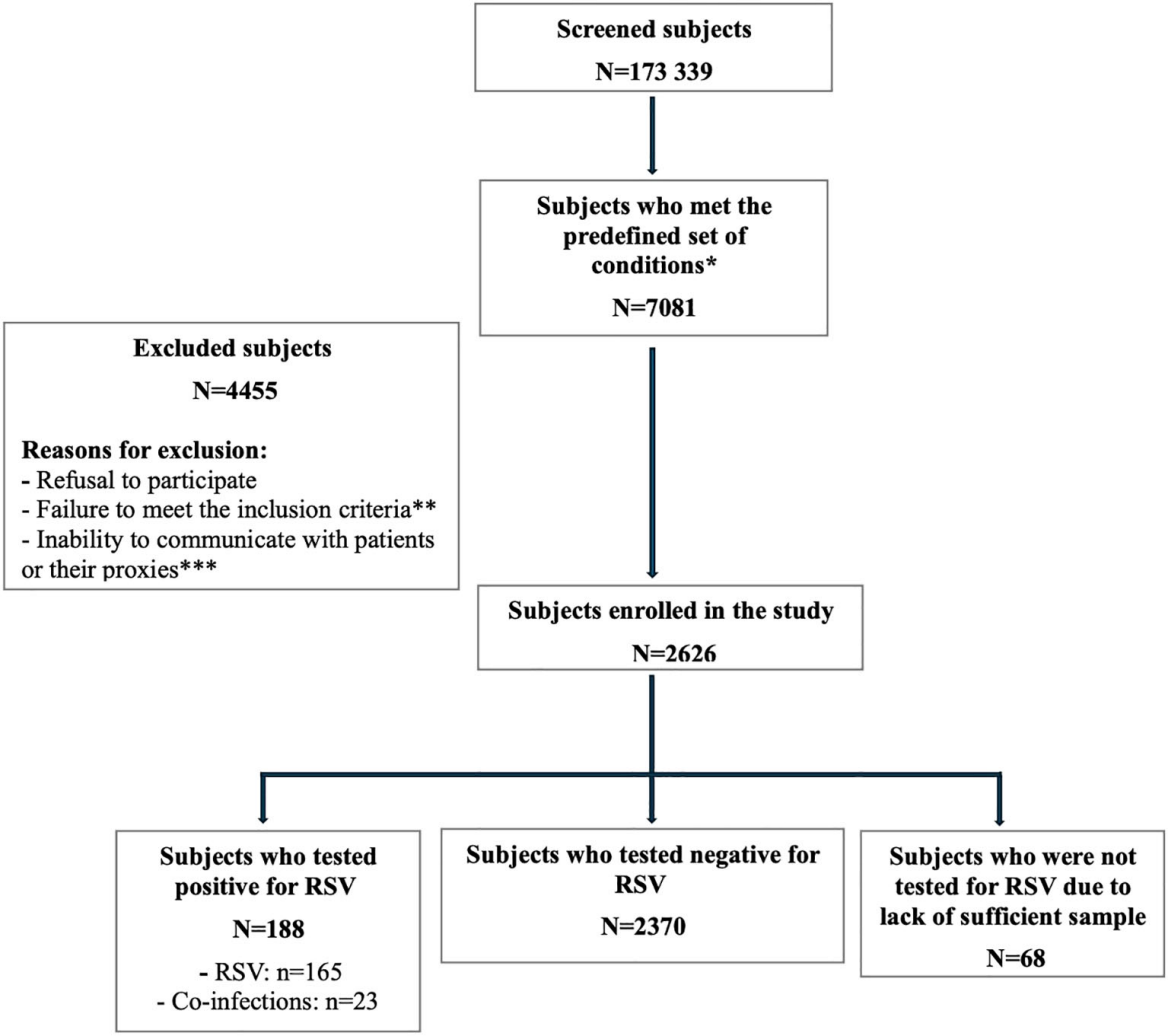
Flow diagram (January 3, 2019 – September 30, 2021). * The predefined set of conditions includes a wide range of admission diagnoses, which serve as eligibility criteria for enrolling subjects aged 5 years and older, as well as those younger than 5 years (see **Supplementary Tables S1**, **S2**). **Inclusion criteria: -Symptoms occurring within seven days or less between the onset of symptoms and the admission to the hospital, for patients younger than 5 years of age. –ECDC modified case definition for influenza like-illness (ILI) in last 7 days (at least one of the four systemic symptoms (fever or feverishness, headache, myalgia or malaise) plus at least one of the four respiratory symptoms (cough, sore throat, shortness of breath or nasal congestion), for patients aged 5 years or older. ***Inability to communicate with patients or their proxies: discharged or deceased or sedated/intubated/child and parents not available at the time of enrollment or lockdown and no direct contact allowed with the patients according to IRB restrictions.

From January 2019 to September 2021, a total of 7081 hospitalized patients met the case definition, with 2626 of them being enrolled in the study. A total of 4,455 subjects were excluded due to refusal to participate, inability to communicate with the patient or proxy, or failure to meet the inclusion criteria, which included symptom onset >7 days before admission (for children <5 years) or not fulfilling the ECDC ILI case definition (for patients ≥5 years). Additional exclusions occurred during COVID-19 lockdown periods when IRB restrictions limited patient contact. Out of the enrolled subjects, 2558 were tested for RSV, out of whom 188 subjects (7.3%) had a positive test result. The remaining 68 of the 2626 enrolled subjects were not tested for RSV because they were already diagnosed with other viral infections and declined to submit an additional sample (**Figure 1**).

The 2020–2021 season had the lowest positivity rate (n= 18, 2.1%) compared to the 2019-2020 (n= 118, 11.1%) and 2018-2019 (n= 52, 8.1%) seasons (p-value <0.001; OR = 0.246 [0.142 – 0.424]). Viral co-infections were detected in 12.2% (n=23) of the 188 RSV-positive subjects (**Supplementary Figure S1**). Due to the small number of detected co-infection cases, no clear seasonal pattern could be identified, although the observed distribution across months is described in **Supplementary Figure S1**.

### 3.2 Demographic and clinical characteristics of subjects

**Supplementary Table S3** summarizes the demographic and general characteristics of RSV-positive and -negative subjects. Of the 188 RSV-positive subjects, 74.5% were below 5 years of age (n=140), 50% below one year of age (n=94), 30.9% below 6 months of age (n=58) and 9.6% aged 65 years and older (n=18). Most of the subjects (n=1,573; 62%) resided in Beirut and Mount Lebanon governorates reflecting the population served by 4 of the 6 sentinel hospitals. Notably, the percentage of RSV-positive subjects in Akkar was higher (n=19, 14.1%) compared to Beirut (n=68; 8.2%) and this was statistically significant (p-value 0.028). There were no significant differences between both RSV and non-RSV groups regarding gender, smoking status, household crowding index, and travel history in the preceding 12 days (**Supplementary Table S3**). Among RSV-positive subjects aged 2 years or younger, 73.8% were breastfed (n=90) compared to 63.4% (n=310) in the RSV-negative group, with this difference being statistically significant (p-value 0.031). Being breastfed did not correlate with decreased severity outcomes (ICU admission, mechanical ventilation, and mortality). Additionally, no differences were observed in the duration of breastfeeding between RSV and non-RSV groups for children aged 2 years or younger. To further evaluate the impact of breastfeeding duration on the risk of RSV infection, we analyzed its effect on children aged between 6 months and one year. The analysis revealed that breastfeeding for 6 months or more provided 50% more protection against RSV infection compared to being breastfed for less than 3 months (OR = 0.449 [0.25-0.82], p-value 0.009).

Among adults aged 65 years and above, 88.9% of the RSV-positive cases (n=16/18) had co-morbidities with 44.4% (n=8/18) presenting with two or more. The most common underlying comorbidities among RSV-positive subjects aged 65 and above (n=18), were cardiovascular disease (72.2%, n=13) and diabetes (27.8%, n=5). There were no significant differences between the RSV-positive and RSV-negative groups (**Supplementary Table S4**).

### 3.3 Clinical presentation and admission diagnoses

The predominant presenting symptoms among all RSV-positive subjects included cough (n=176, 93.6%) (p-value <0.001), followed by fever (n=140, 74.5%) (p-value 0.072), shortness of breath (n=41, 73.2%) (p-value 0.576) and nasal congestion (n=127, 67.6%) (p-value <0.001). Fever, cough, nasal congestion, wheezing, neurological symptoms, diarrhea and decrease in oral intake were significantly associated with RSV infection (p-value < 0.05) whereas fatigue and headache were more significantly associated with non-RSV infections (p-value <0.001) (**Table 2**). When examining children under 5 years and older adults aged 65 years and above separately, we found that cough was significantly associated with RSV-positive cases in both age groups (p-values <0.001 and 0.012, respectively). Nasal congestion was significantly associated with RSV infections in children under 5 years (p-value < 0.001), while fatigue, headache, and abdominal pain were significantly associated with non-RSV infections in this age group (p-value = 0.001, p-value = 0.029, and p-value = 0.020, respectively). In those aged 65 years and above, myalgia, sore throat, nasal congestion, and wheezing were observed more frequently in RSV cases than in non-RSV cases (50%, 27.8%, 44.4%, and 22.2% respectively), though this observation was not statistically significant (**Table 2**).

**Table 2:** Correlation between symptoms and RSV detection in all age groups, children under five years, and older adults 65 years and above.

Symptoms	RSV (All age groups)	p-value	UOR [95%CI]	RSV (Children < 5 years)	p-value	UOR [95%CI]	RSV (older adults ≥ 65 years)	p-value	UOR [95%CI]
Systemic symptoms									
Fever/Feverishness	140/188 (74.5)	0.072		110/140 (78.6)	0.440		7/18 (38.9)	0.251	
Malaise/ fatigue/ lethargy	85/180 (47.2)	**<0.001**	**0.458 [0.338 - 0.621]**	55/132 (41.7)	**0.001**	**0.536 [0.368 - 0.779]**	13/18 (72.2)	0.785*	
Headache	23/124 (18.5)	**<0.001**	**0.434 [0.273 - 0.689]**	5/76 (6.6)	**0.029**	**0.363 [0.141 - 0.936]**	3/18 (16.7)	0.164	
Myalgia	41/92 (44.6)	0.598		15/44 (34.1)	0.211		9/18 (50.0)	0.750	
Respiratory symptoms									
Cough	176/188 (93.6)	**<0.001**	**5.936 [3.286 - 10.725]**	133/140 (95.0)	**<0.001**	**9.907 [4.567 - 21.487]**	18/18 (100)	**0.012**	**1.041 [1.022 - 1.061]**
Sore throat	48/143 (33.6)	0.103		34/95 (35.8)	0.186		5/18 (27.8)	0.344*	
Shortness of breath	41/56 (73.2)	0.576		2/9 (22.2)	0.175*		14/18 (77.8)	0.330*	
Nasal congestion	127/188 (67.6)	**<0.001**	**3.393 [2.472 - 4.656]**	106/140 (75.7)	**<0.001**	**3.029 [2.015 - 4.583]**	8/18 (44.4)	0.112*	
Wheezing	95/188 (50.5)	**<0.001**	**3.047 [2.256 - 4.116]**	81/140 (57.9)	**<0.001**	**2.603 [1.805 - 3.756]**	8/18 (44.4)	0.054*	
Neurological symptoms	41/188 (21.8)	**0.016**	**1.561 [1.084 - 2.246]**	29/140 (20.7)	0.758		4/18 (22.2)	0.325*	
Other symptoms									
Nausea/Vomiting	83/188 (44.1)	0.197		69/140 (49.3)	0.676		4/18 (22.2)	1.000*	
Diarrhea	69/188 (36.7)	**<0.001**	**1.827 [1.338 - 2.494]**	63/140 (45.0)	0.803		0/18 (0.0)	0.092*	
Abdominal pain	35/147 (23.8)	0.730		27/99 (27.3)	**0.020**	**0.571 [0.355 - 0.918]**	1/18 (5.6)	0.334*	
Decrease in PO intake/Dehydration	92/188 (48.9)	**0.004**	**1.543 [1.146 - 2.078]**	78/140 (55.7)	0.133		2/18 (11.1)	0.266*	

RSV, Respiratory Syncytial Virus; n/N, Frequency; %, Percentage; UOR, Unadjusted Odds Ratio; CI, Confidence Interval.

Bold p-values are the significant ones (p-value <0.005).

**Figure 2:**
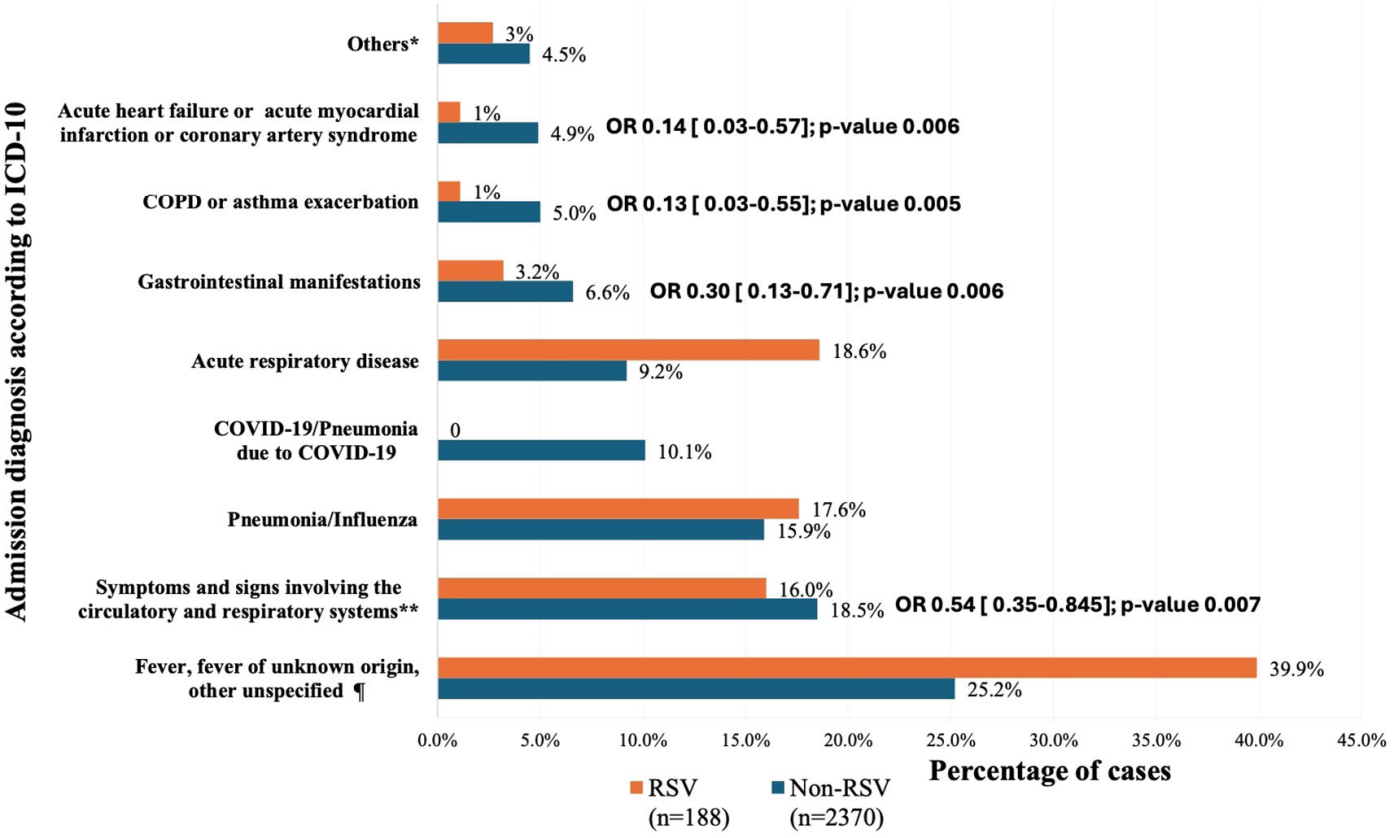
Comparison of the admission diagnoses (according to ICD 10) between RSV-positive and RSV-negative subjects. *Others: Altered consciousness, convulsions, Febrile convulsions (n=43); Sepsis, Systemic inflammatory response (n=32); Metabolic disorders (n=26); Myalgia (n=11). **Symptoms and signs involving the circulatory and respiratory systems: acute respiratory distress or failure, cough, dyspnea or respiratory or breathing abnormality, hypoxemia, or chest symptoms. ¶ “Fever, fever of unknown origin or unspecified” was the reference group. ICD, International Classification of Diseases; COPD, Chronic Obstructive Pulmonary Disease; COVID-19, Coronavirus Disease -2019. ORs represent the associations with RSV positivity. Only statistically significant ORs were included.

**Figure 2** illustrates the comparison of admission diagnoses between RSV-positive and RSV-negative subjects. The most common admission diagnoses in RSV-positive subjects were fever (n=75, 39.9%), acute upper or lower respiratory disease (n=35, 18.6%), pneumonia/influenza (n=33, 17.6%) and symptoms and signs involving the circulatory and respiratory systems (n=30, 16%). Gastrointestinal manifestations, such as abdominal pain/cramping, diarrhea, vomiting, and/or nausea, represented 3.2% of the admission diagnoses in RSV-positive subjects (n=6). The odds of testing positive for RSV was lower in patients presenting with symptoms and signs involving the circulatory and respiratory symptoms, gastrointestinal manifestations, Chronic Obstructive Pulmonary Disease (COPD) or acute asthma or exacerbation or acute heart failure, acute myocardial infarction or coronary artery syndrome, compared to those presenting with fever (p-value <0.005). Subjects with acute respiratory disease had similar odds of testing positive for RSV compared to those with fever, however this was not statistically significant (p-value >0.005).

The most common admission diagnoses among RSV-positive subjects under 5 years of age were fever, acute upper or lower respiratory disease and symptoms and signs involving the circulatory and respiratory symptoms. Conversely, in RSV-positive subjects aged 50 years old and above, pneumonia/influenza was the most common admission diagnosis, followed by acute upper or lower respiratory disease (**Supplementary Figure S2**). Gastrointestinal manifestations were only noted in children under 5 years of age. Similarly, no statistically significant differences were reported.

### 3.4 Risk of RSV-associated ICU admission, mechanical ventilation, and in-hospital death

**Figure 3:**
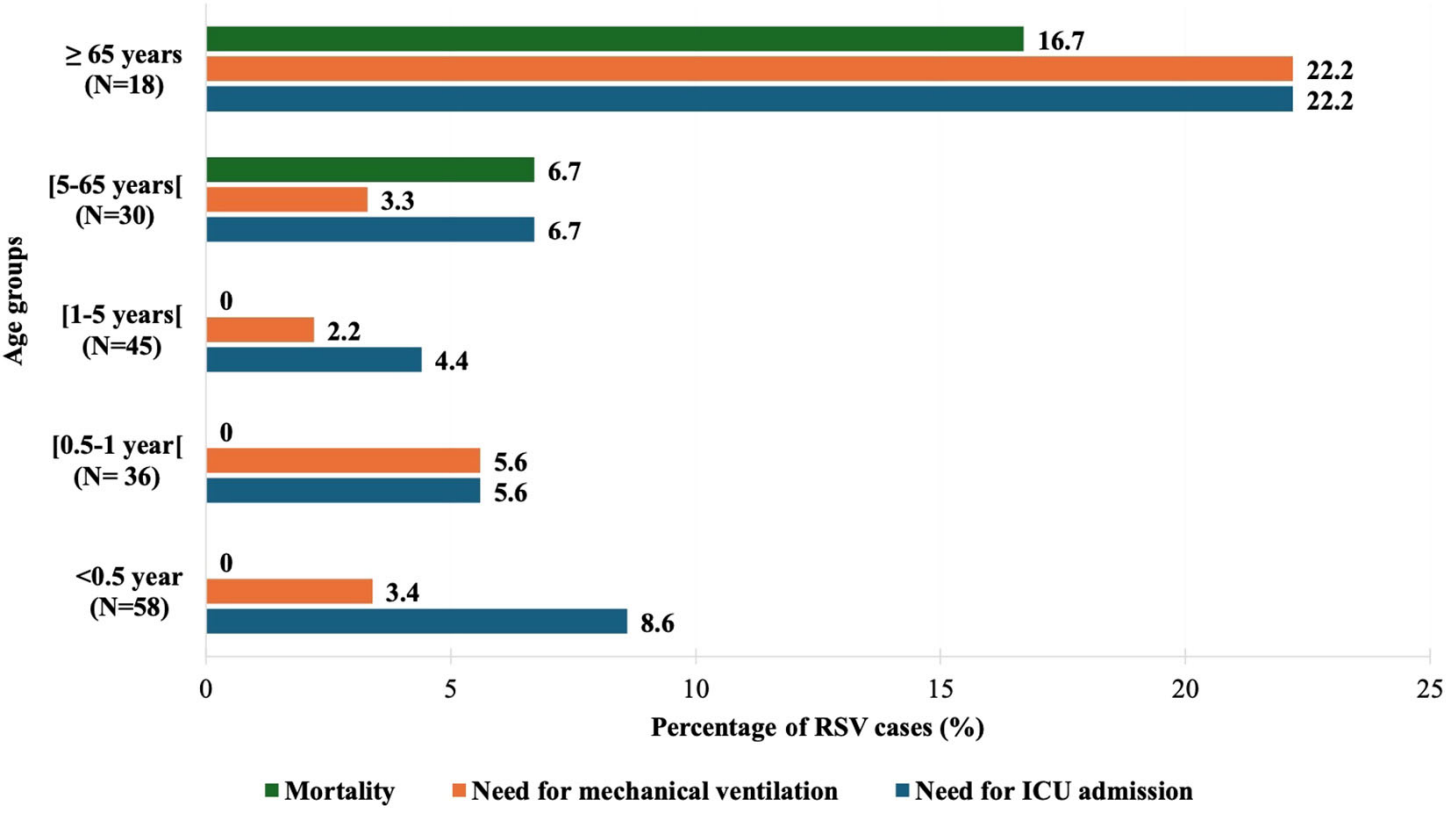
Rate of severe outcomes in RSV-positive patients by age groups. ICU, Intensive Care Unit.

**Figure 4:**
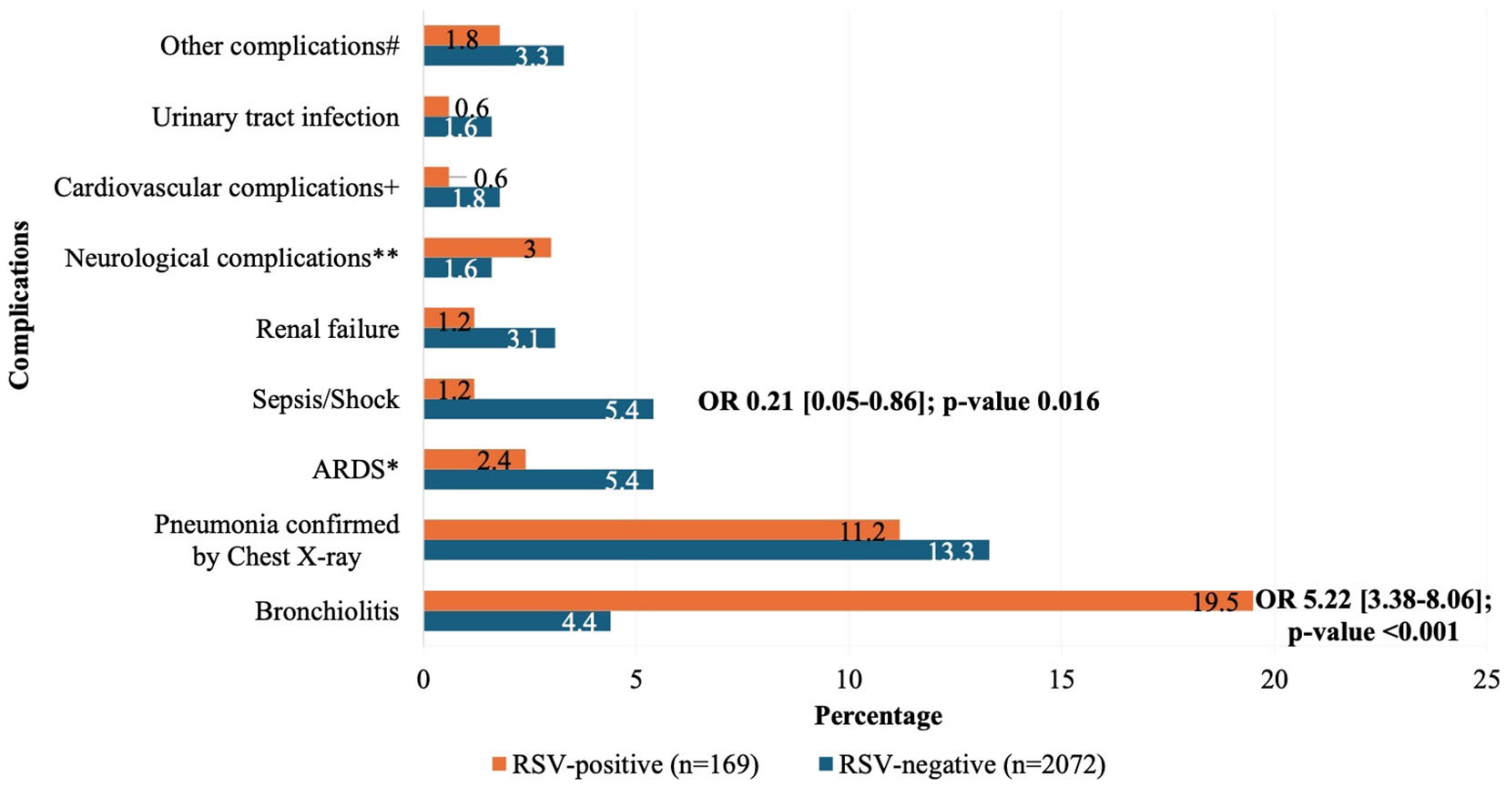
Comparison of the complications during hospital stay in RSV-positive and RSV-negative subjects. *ARDS: Acute respiratory distress syndrome. **Neurological complications: Seizures (n=19), Guillain-Barré syndrome (n=12), encephalopathy/encephalitis (n=9). ^+^Cardiovascular complications: Myocardial infarction (n=20), cardiomyopathy (n=11), stroke (n=4), pericarditis (n=2). ^#^Other complications: Pneumothorax (n=1), pulmonary embolism (n=1), pleural effusion (n=1), hyperglycemia (n=1), hemorrhage (n=1), liver failure (n=1), myositis (n=2), croup (n=15), hemorrhagic pneumonia (n=16). ORs represent the associations with complications. Only statistically significant ORs were included.

Overall, 8% of the RSV-positive subjects required ICU admission (n=15), 5.3% received mechanical ventilation (n=10) and 2.7% died in the hospital (n=5) ICU admissions, longer hospital stay, and mortality were more common among RSV-negative cases (**Table 3**). Severe outcomes were more common among RSV-positive subjects aged 65 years or older; however, this observation was not significant (p-value >0.05) (**Table 4**; **Figure 3**). Bronchiolitis and pneumonia were the most common complications associated with RSV infection (p-value <0.001, OR = 5.22 [3.38 – 8.06] for bronchiolitis) (**Figure 4**). Bronchiolitis occurred only in infants and children less than 5 years of age. Risk factors for RSV-associated ICU admission, mechanical ventilation and mortality are summarized in **Table 4**. The presence of two or more comorbidities significantly increased the odds of mechanical ventilation (OR = 7.44 [1.13-48.99], p-value 0.037) and in-hospital death (OR = 22.67 [1.91-268.50], p-value 0.013). The risks for mechanical ventilation (OR = 8.32 [2.20-31.37], p-value 0.004)) and in-hospital death (OR = 11.50 [1.82-72.85], p-value 0.016)) were higher among RSV-positive subjects with underlying cardiovascular diseases relative to those without cardiovascular diseases. There were no significant differences with respect to age, gender, smoking status, prematurity, and breastfeeding for any of the severity outcomes. RSV-positive subjects requiring oxygen supplementation at presentation had a significantly higher risk of ICU admission, mechanical ventilation, and death (p-value<0.001).

**Table 3:** Severity and outcome of hospitalized patients by RSV infection status.

Severity parameters and mortality	Total n/N (%)	RSV-negative n/N (%)	RSV-positive n/N (%)	p-value	UOR [95%CI]
Severity					
Need for ICU admission	441/2552 (17.3)	426/2365 (18.0)	15/187 (8.0)	**<0.001**	**0.397 [0.232 - 0.680]**
Need for mechanical ventilation	151/2552 (5.9)	141/2365 (6.0)	10/187 (5.3)	0.732	
Oxygen supplementation	719/2488 (28.9)	675/2303 (29.3)	44/185 (23.8)	0.111	
Vasopressor use	160/2543 (6.3)	148/2360 (6.3)	12/183 (6.3)	0.878	
LOS, mean days (± SD) (n=2554)	6.99 (± 7.49)	7.05 (± 7.47)	4.88 (± 4.07)	**<0.001**	**0.932 [0.900 - 0.965]**
ICU duration, mean days (± SD) (n=379)	9.71 (± 8.39)	9.55 (± 8.13)	6.33 (± 5.34)	0.238	
Complications					
No complications	1588/2241 (70.9)	1477/2072 (71.3)	111/169 (65.7)	Ref	
One complication	506/2241 (22.6)	471/2072 (22.7)	50/169 (29.6)	**0.034**	**1.459 [1.028 - 2.070]**
≥ 2 complications	147/2241 (6.6)	140/2072 (6.8)	8/169 (4.7)	0.479	
Mortality	116/2550 (4.5)	111/2363 (4.7)	5/187 (2.7)	0.201	

RSV, Respiratory Syncytial Virus; n/N, Frequency; %, Percentage; UOR, Unadjusted Odds Ratio; CI, Confidence Interval; ICU, intensive care unit; LOS, Length of stay; SD, standard deviation.

Bold p-values are the significant ones (p-value <0.005).

**Table 4:** Risk factors for RSV-associated ICU admission, mechanical ventilation, and in-hospital death.

Baseline characteristics	Non-ICU admission, n/N(%)	ICU admission, n/N(%)	p-value	Non-Mechanical ventilation, n/N (%)	Mechanical Ventilation, n/N (%)	p-value	Recovery, n/N (%)	Death, n/N (%)	p- value
Age groups									
< 0.5 year	53/172 (30.8)	5/15 (33.3)	0.749	56/177 (31.6)	2/10 (20.0)	0.978	58/182 (31.9)	0/5 (0.0)	0.997
[0.5-<1 year]	34/172 (19.8)	2/15 (13.3)	0.851	34/177 (19.2)	2/10 (20.0)	0.669	36/182 (19.8)	0/5 (0.0)	0.998
[1 - <5 years]	43/172 (25.0)	2/15 (13.3)	0.677	44/177 (24.9)	1/10 (10.0)	0.771	45/182 (24.7)	0/5 (0.0)	0.998
[5-<65 years]	28/172 (16.3)	2/15 (13.3)	Ref	29/177 (16.4)	1/10 (10.0)	Ref	28/182 (15.4)	2/5 (40.0)	Ref
≥ 65 years	14/172 (8.1)	4/15 (26.7)	0.134	14/177 (7.9)	4/10 (40.0)	0.069	15/182 (8.2)	3/5 (60.0)	0.287
Gender									
Male	103/172 (59.9)	6/15 (40.0)	Ref	104/177 (58.8)	5/10 (50.0)	Ref	107/182 (58.8)	2/5 (40.0)	Ref
Female	69/172 (40.1)	9/15 (60.0)	0.134	73/177 (41.2)	5/10 (50.0)	0.744*	75/182 (41.2)	3/5 (60.0)	0.651*
Number of comorbidities									
None	129/172 (75.0)	8/15 (53.3)	Ref	134/177 (75.7)	3/10 (30.0)	Ref	136/182 (74.7)	1/5 (20.0)	Ref
One	31/172 (18.0)	5/15 (33.3)	0.114	31/177 (17.5)	5/10 (50.0)	**0.009**	34/182 (18.7)	2/5 (40.0)	0.093
Two or more	12/172 (7.0)	2/15 (13.3)	0.243	12/177 (6.8)	2/10 (20.0)	**0.037**	12/182 (6.6)	2/5 (40.0)	**0.013**
Underlying comorbidities									
Cardiovascular disease	20/172 (11.6)	4/15 (26.7)	0.107*	19/177 (10.7)	5/10 (50.0)	0.004*	21/182 (11.5)	3/5 (60.0)	0.016*
Respiratory disease	8/172 (4.7)	2/15 (13.3)	0.186*	8/177 (4.5)	2/10 (20.0)	0.092*	9/182 (4.9)	1/5 (20.0)	0.243*
Diabetes	6/172 (3.5)	1/15 (6.7)	0.449*	6/177 (3.4)	1/10 (10.0)	0.324*	6/182 (3.3)	1/5 (20.0)	0.175*
Immunosuppression**	6/172 (3.5)	1/15 (6.7)	0.449*	7/177 (4.0)	0/10 (0.0)	1.000*	6/182 (3.3)	1/5 (20.0)	0.175*
Renal disease	1/172 (0.6)	0/15 (0.0)	1.000*	1/177 (0.6)	0/10 (0.0)	1.000*	1/182 (0.5)	0/5 (0.0)	1.000*
Neuromuscular disease	5/172 (2.9)	1/15 (6.7)	0.399*	5/177 (2.8)	1/10 (10.0)	0.284*	6/182 (3.3)	0/5 (0.0)	1.000*
Cirrhosis	2/172 (1.2)	0/15 (0.0)	1.000*	2/177 (1.1)	0/10 (0.0)	1.000*	2/182 (1.1)	0/5 (0.0)	1.000*
Rheumatologic disease	2/172 (1.2)	0/15 (0.0)	1.000*	2/177 (1.1)	0/10 (0.0)	1.000*	2/182 (1.1)	0/5 (0.0)	1.000*
Smoking status of patient or household									
Never smoker	54/165 (32.7)	5/14 (35.7)	Ref	56/169 (33.1)	3/10 (30.0)	Ref	57/174 (32.8)	2/5 (40.0)	Ref
Past or current smoker	111/165 (67.3)	9/14 (64.3)	0.776*	113/169 (66.9)	7/10 (70.0)	1.000*	117/174 (67.2)	3/5 (60.0)	0.665*
Prematurity (≤2 years)	1/115 (0.9)	0/9 (0.0)	1.000*	1/119 (0.8)	0/5 (0.0)	1.000*	1/124 (0.8)	0/5 (0.0)	NA
Breastfeeding (≤2 years)	84/113 (74.3)	6/9 (66.7)	0.696*	88/118 (74.6)	2/4 (50.0)	0.281*	90/122 (73.8)	0/5 (0.0)	NA
Breastfeeding duration (≤2 years)									
< 3 months	36/82 (43.9)	4/6 (66.7)	Ref	39/86 (45.3)	1/2 (50.0)	Ref	40/88 (45.5)	0/5 (0.0)	NA
3 months to < 6 months	32/82 (39.0)	1/6 (16.7)	0.268	32/86 (37.2)	1/2 (50.0)	0.890	33/88 (37.5)	0/5 (0.0)	NA
6 months or more	14/82 (17.1)	1/6 (16.7)	0.704	15/86 (17.4)	0/2 (0.0)	0.999	15/88 (17.0)	0/5 (0.0)	NA
Oxygen supplementation at presentation	31/170 (18.2)	13/15 (86.7)	<0.001*	34/175 (19.4)	10/10 (100)	<0.001*	39/180 (21.7)	5/5 (100)	0.001*
Laboratory proven viral co-infection	22/172 (12.8)	1/15 (6.7)	0.699*	23/177 (13.0)	0/10 (0.0)	0.614*	22/182 (12.1)	1/5 (20.0)	0.485*
Laboratory proven bacterial co-infection	21/159 (13.2)	3/13 (23.1)	0.396*	22/177 (13.4)	2/8 (25.0)	0.309*	24/167 (14.4)	0/5 (0.0)	1.000*

Pearson’s Chi-Square test was used (no expected count less than 5).

*Fisher’s exact test was used when expected count was less than 5.

**Immunosuppression, rheumatologic or autoimmune diseases or malignancies.

n/N: Frequency; %: Percentage. Bold p-values are the significant ones (p-value <0.005).

## 4 Discussion

In this study, RSV was detected in 7.3% of hospitalized patients in Lebanon; 1 in two of the cases being less than one year of age and 1 in 10 being aged 65 year or older. In the MENA region, the RSV infection rates among hospitalized subjects ranged between 4% and 82%. This variation may be due to different case definitions, seasons, or studied populations (Youssef et al., 2021). The lowest rate (4%) was reported on children <14 years with acute respiratory tract infections (al Rashed, 1998), while the highest rate (82%) was found on infants with bronchiolitis (Al Shibli et al., 2016). One third of the RSV positive subjects in our study were below 6 months of age, which is lower than the rate reported by a systematic review on the global, regional, national disease burden estimates of ALRI due to RSV, which estimated that around 45% of hospital admissions due to RSV-ALRI globally occurred in children younger than 6 months of age (Shi et al., 2017). This could be due to the different predefined set of conditions required in our study that included both ALRI and upper respiratory tract infections. Furthermore, a study from the global epidemiology of RSV (GERi) network from 15 countries found that 55.6% of the cases occurred in children less than one year of age and 8% of cases in those aged 65 years and older. This is similar to the percentages reported in our study (Staadegaard et al., 2021).

RSV infection has a wide spectrum of clinical manifestations. In this study, respiratory symptoms (cough, nasal congestion, and wheezing), diarrhea and dehydration were significantly associated with RSV infection whereas fatigue, malaise, lethargy, and headache were more significantly associated with non-RSV infections. A study conducted between 2016 and 2018 on 500 children less than 5 years of age reported that moderate bronchiolitis, fever for more than 4 days, cough, headache, dyspnea, conjunctivitis, tachypnea, diarrhea, fatigue, severe dehydration and nasal congestion were significantly associated with RSV infection (Divarathna et al., 2023). Similar to our results, a study by Belongia et al. on adults ≥60 years old reported that common symptoms of RSV included sore throat, cough, myalgia and wheezing (Belongia et al., 2018). It is important to note that 3.2% and 1% of our RSV-positive subjects presented with gastrointestinal manifestations and acute heart failure, respectively, upon admission. Gastrointestinal symptoms are not uncommon manifestations of acute viral respiratory infections, including influenza and RSV (Minodier et al., 2017). A study conducted by Hijazi et al. in 2022 described the detection of RSV RNA in the stools of children presenting with gastroenteritis. Yet, there is no evidence of direct replication of RSV in the intestinal tissue and the diarrhea, might be attributed to inflammation or dysregulation of the intestinal microbiota (Hijazi et al., 2022). Healthcare providers should be aware of these extrapulmonary manifestations of RSV as they may result in unexpected clinical deterioration and present significant diagnostic and therapeutic challenges (Eisenhut, 2006; Gkentzi et al., 2018).

Infants and children are universally exposed to RSV. Breastfeeding, especially for the first 6 months, is known to serve as the nature’s first vaccine during the most vulnerable period of contracting RSV infection (Mineva et al., 2023). However, our study showed that breastfeeding was more common among RSV-positive subjects, thereby identifying it as a risk factor for RSV, which is in line with a study published by Pandolfi et al (Pandolfi et al., 2019). One possible explanation for this finding is that breastfeeding may serve as a proxy for closer contact of the infant with the mother as well as other household members. The benefits of breastfeeding are mainly related to its duration as it modulates the immune system through the high levels of IgA class antibodies (Ferreira et al., 2022). In our study, the impact of breastfeeding duration was better assessed by focusing on infants aged between 6 months and one year, since infants aged less than 6 months may have not had sufficient time to develop an optimal immune response and thus may be less protected against viral infections. Our analysis revealed that breastfeeding for 6 months or more conferred better protection against RSV in this age group. This result is consistent with the results of Pandolfi et al. that confirmed the time-dependent effect of exclusive breastfeeding on the protection against viral infections (Pandolfi et al., 2019).

Regarding the complications of RSV, our analysis revealed that 8% of the RSV-positive subjects required ICU admission, 5.3% received mechanical ventilation and 2.7% died. These findings were within range of the data from the MENA region where the studies on RSV showed that ICU admissions ranged between 1% and 15%, the need for mechanical ventilation ranged between 1% and 10% and the overall RSV -related mortality across all age groups was 1.9% (Youssef et al., 2021). Our analysis showed that the risk factors for severe RSV outcomes among RSV-positive patients included having ≥ one underlying comorbidity, in particular cardiovascular disease. Wang et al. conducted a systematic review and meta-analysis on the risk factors for ALRI caused by RSV in preterm infants and young children and reported that underlying medical conditions namely congenital heart diseases was consistently associated with higher risk of severe outcomes (longer hospital stay, supplemental oxygen administration, and mechanical ventilation or intensive care unit admission) (Wang et al., 2024). On the other hand, a recent systematic review among adults in the United States, Europe, East Asia, and New Zealand identified older age (≥ 65 years), chronic cardiac and pulmonary disease, chronic kidney disease, diabetes mellitus, immunocompromised status, socioeconomic level, and nursing home residence as risk factors correlating with more severe RSV outcomes in adults. These outcomes included hospitalization, ICU admission, emergency department visits, re-consultation with new or worsened symptoms, requirement of mechanical/noninvasive ventilation or vasopressor support, mortality, pneumonia, myocarditis, and encephalitis (Njue et al., 2023). Underlying chronic pulmonary disease and functional disability in elderly adults aged 65 years and above were shown to independently increase the risk of hospitalization due to RSV infection (Walsh et al., 2004).

RSV typically follows a clear seasonal pattern, with cases rising as temperatures decline. This trend could be attributed to increased viral transmission due to several factors including more frequent indoor gatherings during colder months, increased viral stability and host susceptibility to infections (Noyola and Mandeville, 2008; Kaler et al., 2023). However, the COVID-19 pandemic has reportedly disrupted the RSV seasonality and altered its transmission (Li et al., 2024). In our study, we observed a 10-fold decrease in the RSV positivity rate following the introduction of COVID-19-related non-pharmaceutical interventions such as masking, school closures, and social distancing measures. However, after alleviating these interventions in the summer of 2021, we recorded a notable off-seasonal increase in the RSV-positive cases. This surge occurred approximately 20 to 24 weeks after the originally anticipated peak. Comparable patterns were observed globally, with delays in the expected RSV season ranging from 13 weeks in France to 88 weeks in South Korea (Rios-Guzman et al., 2024). Moreover, according to the CDC, positivity rate of RSV in the United States (US) failed to reach 1% during the typical 2020–2021 season, and following lifting of the NPIs in the summer of 2021, a resurgence in RSV cases was noted around 32 weeks after the originally anticipated peak (Rios-Guzman et al., 2024). This atypical inter-seasonal resurgence of respiratory infections around the world may be explained by the “immunity debt” as described by the Pediatric Infectious Disease Group, due to the lack of immune stimulation and decreased natural immunity which led to the off-season increase in RSV activity once the NPIs were relaxed (Cohen et al., 2021; Piralla et al., 2023).

The strengths of this study include its prospective multicenter design with a standardized patients screening method across all participating sites, the centralized confirmation of respiratory viruses at CIDR, the reference center, and the extended study period covering three consecutive seasons. In addition, this study highlights the RSV epidemiology, risk factors, and seasonality across all age groups in Lebanon, including a large number of hospitalized adults meeting the inclusion criteria, with a wide range of clinical presentations and medical conditions. Nonetheless, certain limitations must be recognized. First, the recruitment was limited solely to hospitalized patients, which may limit the generalizability of the results to reflect the RSV disease presentation and incidence in the outpatient settings. Second, the RSV positivity rate may be underestimated in older adults since they frequently have delayed or atypical clinical presentations with lower viral loads in nasal secretion when compared to infants and children, which can further decrease the sensitivity of the diagnostic test and may underestimate the true burden of RSV in the older population (Kim and Choi, 2024).

## 5 Conclusion

RSV imposes a substantial disease burden across all age groups in Lebanon, particularly among infants, older adults, and individuals with underlying comorbidities. Severe outcomes—including ICU admission, mechanical ventilation, and in-hospital death—were more frequent in older adults and in patients with cardiovascular disease or multiple comorbidities. The COVID-19 pandemic disrupted typical RSV seasonality, followed by an off-season resurgence, highlighting the need for continuous surveillance to monitor epidemiology and disease burden.

These findings provide critical evidence to inform public health strategies, including the implementation of RSV vaccines and monoclonal antibodies, and to guide policy decisions in the MENA region.

## Data Availability

The raw data supporting the conclusions of this article will be made available by the authors, without undue reservation.
