# Supplementary material for: Epidemiologic characteristics and clinical outcomes of respiratory syncytial virus in hospitalized care in Lebanon: a prospective observational study
Source: Front Cell Infect Microbiol. 2026 Jan 7;15:1711410. doi: 10.3389/fcimb.2025.1711410 (PMC12819600; doi:10.3389/fcimb.2025.1711410)
Supplement: Supplementary file 1 [file Table1.docx]

**Supplementary material**

**Table 1S. Case ascertainment/Case finding: Admission diagnoses**. **Admission diagnoses possibly associated with an influenza infection for patients 5 years old or older.**

| **For patients 5 years and older** | **ICD 9 Codes** | **ICD 10 Codes** |
| --- | --- | --- |
| Acute upper or lower respiratory disease | 382.9; 460-466 | J00-J06, J20-J22,  H66.90 |
| Acute myocardial infarction or acute coronary syndrome | 410-411 and 413-  414 | I20-I25.9 |
| Acute asthma or exacerbation | 493.92 | J45.901 |
| Acute Heart failure | 428-429.0 | I50-I50.9; I51.4 |
| Pneumonia and influenza | 480-488 | J09-J18 |
| Bronchitis and exacerbations of Chronic  Pulmonary Obstructive disease | 490, 491.21 and  491.22, | J40; J44.0; J44.1 |
| Acute respiratory failure | 518.82 | J96 |
| Myalgia | 729.1 | M79.1 |
| Acute metabolic failure (diabetic coma, renal dysfunction, acid-base disturbances, alterations to the water balance) | 250.1- 250.3; 584-  586; 276-277 | E11.9, E10.9,  E11.65, E10.65,  E10.11, E11.01,  E10.641, E11.641,  E10.69, E11.00,  E10.10, E11.69,  N17.0, N17.1,  N17.2, N17.8,  N17.9, N18.1,  N18.2, N18.3,  N18.4, N18.5,  N18.6M N18.9, N19,  E87.0, E87.1, E87.2,  E87.3, E87.4, E87.5,  E87.6, E87.70,  E87.71, E87.79,  E86.0, E86.1 |
| Altered consciousness, convulsions, febrile convulsions, syncope and collapse | 780.01-780.02;  780.09; 780.2;  780.31-780.32 | R40.20, R40.4,  R40.0, R40.1, R55,  R56.00, R56.01 |
| Dyspnea/respiratory abnormality | 786.0 | R06.0, R06-R06.9 |
| Respiratory abnormality | 786.00 | R06.9 |
| Shortness of breath | 786.05 | R06.02 |
| Respiratory abnormality not otherwise specified | 786.09 | R06.3, R06.00,  R06.09, R06.83 |
| Respiratory symptoms/chest symptoms | 786.9 | R06.89 |
| Fever or fever unknown origin or non-specified | 780.6-780.60 | R50, R50.9 |
| Cough | 786.2 | R05 |
| Sepsis, Systemic inflammatory response syndrome | 995.90-995.94 | R65.10, R65.11,  R65.20, A41.9 |

**Table 2S. Admission diagnoses possibly associated with an influenza infection for the very young pediatric population (0 to less than five years of age)**

| **For patients less than 5 years** | **ICD 9 Codes** | **ICD 10 Codes** |
| --- | --- | --- |
| Acute upper or lower respiratory disease | 382.9; 460 to 466 | J00-J06, J20-J22 |
| Dyspnea, breathing anomaly, shortness of breath, tachypnea (polypnea) | 786.0; 786.00;  786.05-786.07;  786.09; 786.9 | R06.0, R06, R06.9,  R06.3, R06.00,  R06.09, R06.83,  R06.02, R06.82,  R06.2, R06.89 |
| Acute asthma or exacerbation | 493.92 | J45.901 |
| Pneumonia and influenza | 480 to 488 | J09-J18 |
| Acute respiratory failure | 518.82 | J96 |
| Acute heart failure | 428-429.0 | I50-I50.9; I51.4 |
| Myalgia | 729.1 | M79.1 |
| Altered consciousness, convulsions, febrile convulsions | 780.01-780.02; 780.09; 780.31-  780.32 | R40.20, R40.4,  R40.0, R40.1,  R56.00, R56.01 |
| Fever or fever unknown origin or non specified | 780.6-780.60 | R50, R50.9 |
| Cough | 786.2 | R05 |
| Gastrointestinal manifestations | 009.0; 009.3 | A09.0; A09.9 |
| Sepsis, Systemic inflammatory response syndrome, not otherwise specified | 995.90-995.94 | R65.10, R65.11,  R65.20, A41.9 |
| Nausea and vomiting | 078.82; 787.0;  787.01-787.03 | R11; R11.0; R11.10  - R11.12; R11.2 |

**Figure 1S. Frequency of RSV infections and viral co-infections during the 3 seasons of the study.**

*The recruitment during the first season 2018-2019 started in January 2019 due to some delays in study approval.

RSV: Respiratory Syncytial Virus; HRV/EV: Human rhinovirus/enterovirus; HAdV: Human adenovirus; NT: non-typeable.

**Table 3S. Demographics and baseline characteristics of enrolled subjects.**

|  | **RSV-negative**  **n/N (%)** | **RSV-positive**  **n/N (%)** | **p-value** |
| --- | --- | --- | --- |
| **Age groups** |  |  |  |
| < 0.5 year | 196/2364 (8.3) | 58/188 (30.9) | **<0.001** |
| [0.5-1 year[ | 164/2364 (6.9) | 36/188 (19.1) | **<0.001** |
| [1-5 years[ | 441/2364 (18.7) | 46/188 (24.5) | **<0.001** |
| [5-18 years[ | 168/2364 (7.1) | 7/188 (3.7) | 0.489 |
| [18-50 years[ | 434/2364 (18.4) | 13/188 (6.9) | Ref |
| [50-65 years[ | 365/2364 (15.4) | 10/188 (5.3) | 0.834 |
| ≥ 65 years | 596/2364 (25.2) | 18/188 (9.6) | 0.982 |
| **Gender** |  |  |  |
| Male | 1339/2370 (56.5) | 110/188 (58.5) | Ref |
| Female | 1031/2370 (43.5) | 78/188 (41.5) | 0.592 |
| **Residence (N=2458)** |  |  |  |
| Beirut (N=833) | 765/833 (91.8) | 68/833 (8.2) | Ref |
| Mount Lebanon (N=740) | 697/740 (94.2) | 43/740/5.8) | 0.070 |
| North (N=317) | 282/317 (89.0) | 35/317 (11.0) | 0.128 |
| Bekaa (N=311) | 297/311 (95.5) | 14/311 (4.5) | **0.035** |
| Akkar (N=135) | 116/135 (85.9) | 19/135 (14.1) | **0.028** |
| South (N=69) | 66/69 (95.7) | 3/69 (4.3) | 0.267 |
| Other** (N=53) | 51/53 (96.2) | 2/53 (3.8) | 0.264 |
| **Smoking status of patient or household** |  |  |  |
| Never smoker | 785/2332 (33.7) | 60/180 (33.3) | Ref |
| Past or current smoker | 1547/2332 (66.3) | 120/180 (66.7) | 0.928 |
| **Household crowding index (HCI)** |  |  |  |
| Less than or equal to 1 person/room | 1133/2110 (53.7) | 82/148 (55.4) | Ref |
| More than 1 person/room | 977/2110 (46.3) | 66/148 (44.6) | 0.687 |
| **Working in a healthcare facility** | 130/2322 (5.6) | 11/185 (5.9) | 0.844 |
| **Breastfeeding (≤2 years)** | 310/489 (63.4) | 90/122 (73.8) | **0.031** |
| **Breastfeeding duration (≤2 years)** |  |  |  |
| Less than 3 months | 134/300 (44.7) | 40/88 (45.5) | Ref |
| 3 months to less than 6 months | 87/300 (29.0) | 33/88 (37.5) | 0.379 |
| 6 months or more | 79/300 (26.3) | 15/88 (17.0) | 0.176 |
| **Presence of sick contacts (n=2358)** | 944/2172 (43.5) | 105/186 (56.5) | **0.001** |
| **Travel in the last 14 days** | 59/2204 (2.7) | 2/188 (1.1) | 0.230 |

Pearson’s Chi-Square test was used (no expected count less than 5)

*Fisher’s exact test was used when expected count was less than 5

**Other: Baalbeck-Hermel (n=20), Nabatiyeh (n=16), not resident in Lebanon (n=17).

RSV: Respiratory Syncytial Virus; n/N: Frequency; %: Percentage.

**Table 4S. Presence of underlying comorbidities among RSV-positive vs negative cases**

| **Underlying comorbidities** | **[0-5 years [ (N=937)** | | | **[5-65 years [ (N=984)** | | | **≥65 years (N=612)** | | |
| --- | --- | --- | --- | --- | --- | --- | --- | --- | --- |
|  | **RSV-negative  (N=797)**  **n (%)** | **RSV-positive  (N=140)**  **n (%)** | **p-value** | **RSV-negative  (N=954)**  **n (%)** | **RSV-positive  (N=30)**  **n (%)** | **p-value** | **RSV-negative (N=594)**  **n (%)** | **RSV-positive (N=18)**  **n (%)** | **p-value** |
| **Any comorbidity** | 192 (24.1) | 18 (12.9) | **0.003^¶^** | 510 (53.5) | 17 (56.7) | 0.729 | 525 (88.4) | 16 (88.9) | 1.000* |
| **Cardiovascular disease** | 24 (3.0) | 4 (2.9) | 1.000* | 235 (24.6) | 7 (23.3) | 0.871 | 404 (68) | 13 (72.2) | 0.706 |
| **Respiratory disease** | 37 (4.6) | 5 (3.6) | 0.572 | 152 (15.9) | 4 (13.3) | 1.000* | 171 (28.8) | 2 (11.1) | 0.101 |
| **Diabetes** | 4 (0.5) | 0 (0.0) | 1.000* | 123 (12.9) | 2 (6.7) | 0.414* | 216 (36.4) | 5 (27.8) | 0.455 |
| **Immunosuppression**** | 30 (3.8) | 1 (0.7) | 0.071 | 105 (11.0) | 4 (13.3) | 0.565* | 52 (8.8) | 2 (11.1) | 0.668* |
| **Renal disease** | 9 (1.1) | 0 (0.0) | 0.370* | 33 (3.5) | 0 (0.0) | 0.620* | 80 (13.5) | 1 (5.6) | 0.492* |
| **Neuromuscular disease** | 15 (1.9) | 4 (2.9) | 0.510* | 32 (3.4) | 1 (3.3) | 1.000* | 57 (9.6) | 2 (11.1) | 0.689* |
| **Cirrhosis** | 2 (0.3) | 0 (0.0) | 1.000* | 21 (2.2) | 2 (6.7) | 0.153* | 7 (1.2) | 0 (0.0) | 1.000* |
| **Rheumatologic disease** | 2 (0.3) | 0 (0.0) | 1.000* | 29 (3.0) | 0 (0.0) | 1.000* | 30 (5.1) | 2 (11.1) | 0.241* |

Pearson’s Chi-Square test was used (no expected count less than 5)

**^¶^** UOR= 0.465, 95%CI= [0.276 - 0.783]

*Fisher’s exact test was used when expected count was less than 5

** Immunosuppression: rheumatologic or autoimmune diseases or malignancies

RSV: Respiratory Syncytial Virus; n/N: Frequency; %: Percentage.

**Figure 2S. The distribution of admission diagnoses by age group in RSV-positive subjects.**

Symptoms and signs involving the circulatory and respiratory systems: acute respiratory distress or failure, cough, dyspnea or respiratory or breathing abnormality, hypoxemia or chest symptoms

*Others: Altered consciousness, convulsions, febrile convulsions (n=43); Sepsis, Systemic inflammatory response (n=32); Metabolic disorders (n=26); Myalgia (n=11).

COPD: Chronic Obstructive Pulmonary Disease; COVID-19: Coronavirus Disease -2019
